# A portrait of online gambling: a look at a transformation amid a pandemic

**DOI:** 10.1186/s12954-025-01265-1

**Published:** 2025-08-06

**Authors:** Sylvia Kairouz, Annie-Claude Savard, W. Spencer Murch, Melanie Rose Dixon, Nadine Blanchette Martin, Magaly Brodeur, Sophie Dauphinais, Francine Ferland, Denis Hamel, Magali Dufour, Martin French, Eva Monson, Valérie Van Mourik, Adèle Morvannou

**Affiliations:** 1 Concordia University, 1455 de Maisonneuve Blvd. W, H-1125-31, Montréal, H2G 1M8 Québec Canada; 2 Université Laval, Québec City, Québec Canada; 3 Service de recherche en dépendance du CIUSSS de la Capitale-Nationale et du CISSS de Chaudière-Appalaches, Québec City, Québec Canada; 4 Université de Sherbrooke, Sherbrooke, Québec Canada; 5 Université du Québec à Montréal, Montréal, Québec Canada; 6 CIUSSS Centre-Sud-de-l’Île-de-Montréal - Montreal Addiction Rehabilitation Centre, Montréal, Québec Canada

**Keywords:** Gambling, Online gambling, COVID-19 pandemic, Mixed methods design, Population survey, Lived experience

## Abstract

**Background:**

The COVID-19 pandemic brought about an extraordinary societal context in which the gambling offer was modified to meet public health measures intended to curb viral transmission. With many land-based gambling venues being forced to close, gambling opportunities were left almost exclusively to the online domain, thus possibly instigating changes in the population’s online gambling habits. Using a sequential mixed methods design, this study aimed to (1) investigate the self-reported changes in gambling habits of adults in the province of Québec (Canada) following the declaration of the COVID-19 pandemic and ensuing public health responses, and (2) report on their lived experiences of these changes during the first year of the pandemic.

**Method:**

A population survey was conducted with a representative sample of 4,676 online gamblers residing in the province of Québec, which was selected through random digit dialing for telephone interviews and from a web panel. From the initial sample, 96 online gamblers were recruited for in-depth semi-structured interviews inquiring about their gambling experiences during the first year of the pandemic.

**Results:**

The prevalence of online gambling was estimated at 15.6–20.3% of Québec’s population in 2021, among which 5.6% gambled online for the first time during the pandemic, which represented a substantial addition to the 14.7% of people who gambled online both before and during the pandemic. Only 1.4% of people quit online gambling during the pandemic. The impact of the pandemic was similar for frequency, expenditure, and time spent on various online gambling activities, with day trading having increased most during the pandemic. Seeking to earn money was one of several motivations endorsed by participants who had begun or increased online gambling practices during the first year of the pandemic.

**Conclusion:**

The COVID-19 pandemic clearly revealed a significant increase in online gambling practices when changes in the gambling landscape and in daily life occurred due to the health crisis. This calls for a greater attention to the need for comprehensive regulatory measures and a support system for online gambling in a context of a steadily increasing lucrative market.

## Introduction

The emergence of Coronavirus Disease 2019 (COVID-19) and the ensuing public health response have strongly impacted the health and day-to-day lives of people across the globe. Numerous public health measures, which were implemented to contain viral transmission, also limited social gatherings and the operation of nonessential commerce [1]. Worldwide, the context of this rare public health crisis put a spotlight on online gambling while providing a global, naturalistic experiment to understand the impacts of unprecedented changes in the gambling landscape on gambling practices and problems.

In the province of Québec, Canada, the unfurling of pandemic-related public health measures in March 2020 (i.e., multiple lockdowns, curfew, commerce and services being reduced or closed) set the scene for economic instability and uncertainty as people saw their work hours reduced or lost employment altogether depending on whether their work was deemed essential for the health, well-being and safety of Québécois people. The lockdown and social distancing policies in effect at the time also gave rise to various emotional experiences which impacted gambling behaviours, including feelings of boredom, anxiety and loneliness [2].

The province of Québec was no exception in its response to the health crisis in the gambling sector. The pandemic-related public health measures translated into the closure of all legalized land-based gambling venues such as casinos, bingo and electronic gambling halls, and lottery ticket counters, and forced the halt of sports betting activity as sporting events were also suspended [3]. With usual gambling venues unavailable, many people who gamble were faced with a choice in response to this then-new reality: reducing or stopping gambling or migrating to new or alternate gambling platforms (e.g., online lotteries, poker or slot machines). As in other countries, Québec has also seen a general decrease in gambling participation following the emergence of COVID-19, as evidenced by a decline of 48.6% in the total revenue of the State monopoly, Loto-Québec, and 66.2% in their total net income for the 2020–2021 fiscal year [4]. Studies conducted in New Zealand, Australia, Italy, Switzerland, Sweden, Poland and the United Kingdom also show general decreases in gambling participation [5–11]. In Canada, a pre-post pandemic study found that one third of gamblers ceased gambling entirely six months after the pandemic was declared [12].

While engagement in land-based gambling appears to have decreased during the initial phase of the pandemic, the scenario regarding online gambling during the pandemic did not follow quite as clear a trend. To date, studies in multiple jurisdictions have shown that online gambling platforms saw an increased engagement suggesting an influx of new users [13–15]. In Australia, Jenkinson et al. [16] reported a 16% increase in online gambling participation during the pandemic, as well as a 9% increase in respondents who reported gambling at least four times weekly. Of these participants, one third reported creating a new online account during the pandemic. Similar evidence has accumulated in Canada: studies conducted in the province of Ontario revealed a large-scale migration towards online gambling [15, 17] and a survey from British Columbia showed an increase from 22 to 31% in participation rate for online gambling activities since the onset of the pandemic [14]. In this vein, the decline of revenues for Loto-Québec were mirrored by an increase of 171% of revenues generated from their online gambling website (Espacejeux.com) in 2020–2021, thus showing a net expansion of online gambling on this platform during the first year of the pandemic [4]. However, participation in online gambling activities decreased for sports bettors due to the cancellation of sports events [18–21], and other studies highlighted a lack of change altogether in gambling habits despite having migrated from offline to online gambling platforms [22–24].

During the first few months of the pandemic, there seem to have been variations in gambling advertisement in the province. A study having focused on gambling advertisement on social media (i.e., Facebook, Instagram, Twitter) aired by four gambling operators (Loto-Québec, Pokerstars, 888poker, William Hill) shows that advertisement briefly increased in March 2020, i.e., shortly after the World Health Organization’s declaration of a state of public health emergency [25]. From then until August 2020, there were periods of increase and decrease in advertisement, which appeared to coincide with variations in social distancing and public health measures in effect at the time. While the state monopoly (Loto-Québec) used social media platforms such as Facebook and Instagram to inform users about the closing and the gradual opening of their physical gambling venues and aired the least advertisements overall, some of the offshore online operators, deemed illegal in Canada, intensified the advertisement of their online gambling products by airing the most publicity messages during this time or using the pandemic as a promotional tool for their gambling offer.

From a public health perspective, the pandemic-related increase in online gambling participation has raised concerns about the adverse effects of such a shift, particularly for those at risk of problems who migrated to online gambling platforms [26] at a time when available preventive measures remained limited for online gambling. Some evidence suggests that this occurred: in several studies, people who engage in riskier or problematic gambling behaviours were more likely than lower-risk people to migrate towards online gambling or increase their gambling participation during the pandemic [5, 7, 13, 20, 27–32]. In addition, the risk of harm for those considered at-risk may be aggravated by the use of online gambling platforms: when compared to land-based alternatives, online gambling activities are typically accessible at all hours of the day and from virtually any place due to their portability (namely via mobile devices), less supervised by staff with harm reduction training, and have traditionally seen disproportionately high rates of people experiencing harm, among other things due to the fast-paced nature of the online gambling environment and its rapidly-evolving and complex offer [33–35].

In this context, it has been argued that harm reduction pertaining to online gambling is insufficient, requiring a more tailored, multifaceted approach that takes into account the idiosyncrasies of online gambling and its environment. Harm reduction measures, which are largely based on an individually focused responsible gambling approach, do not take into account the broader socio-cultural level, which requires measures to regulate gambling offerings, operators’ practices and the implementation of laws and policies governing them [34, 36]. It has been argued that harm reduction pertaining to online gambling requires further empirical attention, which points to the relevance of conducting a specific investigation of the habits of online gamblers during the COVID-19 pandemic, when online gambling was made more accessible and visible to the population.

Beyond the mere impacts of the pandemic on gambling behaviour, studies have also shed light on the reasons underlying such changes. People who reported reducing their gambling evoked reasons such as financial constraints [6, 7, 37], limited access to gambling venues [7], relational dimensions such as not wanting to gamble in the presence of family members, feeling that they gamble too much, or having been told that they should gamble less [6], and a lack of desire to gamble [37]. Alternatively, reasons reported for increasing online gambling were the unavailability of usual gambling venues [6, 38], boredom [6], financial concerns or seeking to win money [6, 7, 37], stress relief or needing to relax [6, 7, 38], socialization opportunities [6], and having more free time or replacing unavailable leisure activities [7, 38]. In addition to these reasons, our own study revealed that participants also mentioned some attributes related to the online gambling ecosystem including the operators’ practices as reasons for augmenting their online gambling practices. They referred to factors such as operators’ strategies of building “customer” loyalty, advertisement and promotion as well as the accessibility, instantaneity and diversification of online gambling games [38].

Up till now, most studies reporting on the impact of the COVID-19 pandemic have been conducted with convenience samples, and research utilising representative samples remain scarce [39]. Representative samples are vital to provide accurate prevalence estimates for the general population which are needed for policy recommendations and for guiding prevention initiatives and help services. Furthermore, existing knowledge on the impact of the pandemic has been largely produced by quantitative studies. In such an unprecedented context, the contribution of qualitative data focusing on gamblers’ lived experiences is crucial to deepen our understanding of this otherwise rare societal phenomenon. In response to this need, this study used a mixed methods design to investigate the impacts of the COVID-19 pandemic among a representative sample of online gamblers in the province of Québec. More specifically, this study aimed to (1) generate a portrait of changes in online gambling participation during the first year of the pandemic, (2) investigate the impacts of the pandemic on online gambling practices across various activities, and (3) examine the reasons people report for having changed their online gambling habits.

## Method

A sequential mixed methods design was used to achieve the objectives of this study. First, a population study was conducted to generate a portrait of gambling practices of adult online gamblers and assess the impacts of the COVID-19 pandemic. Second, qualitative semi-structured interviews were conducted with a sub-sample of online gamblers to gather information on their lived experiences during the pandemic, including any changes in their practices and the reasons for such changes. Qualitative data were used to inform the main quantitative component of this paper.

### Survey component

#### Study design and participants

Quantitative data were derived from the first wave of the longitudinal study [40], which aimed to document the impact of the COVID-19 pandemic on gambling habits. The target population consisted of non-institutionalized adult (18 years or older) online gamblers who gambled during the 12 and or 24 months preceding the survey, who spoke French or English and lived in private households throughout the province of Québec. The study employed a hybrid sampling technique to reach participants either by telephone or through a web panel.

##### Telephone sample

A probabilistic telephone-based sample was generated to produce estimates of online gambling prevalence and practices in the adult population of Québec. The sampling list included cellular phone users to compensate for the decreasing response rates observed among people reached via landline phones, and landline ownership biases observed for young adult and low-income respondents [41]. Representation among these groups was particularly important for the estimation of gambling participation, as online gambling is disproportionately popular among young adults [42].

Data were collected using computer-assisted telephone interviews over three periods (May to June 2021, July to August 2021, and September to October 2021). Private household (66%) and cellular phone (34%) numbers were selected by random digit dialing. The sample was stratified geographically according to the seven distinct regions along the rural-urban continuum based on Statistics Canada’s classifications. Consenting respondents were screened for online gambling participation, and only those who reported online gambling after February 2019 were retained for the remainder of the interview. Of those contacted via the estimated 162,126 valid phone numbers, 15,817 (9.8%) agreed to participate and reported valid responses to the online gambling participation screener. The final sample consisted of 1,381 participants (8.7%) after excluding a large proportion of ineligible non online gambling participants, and 8 participants who had incomplete answers on the gambling participation questions. Participants were compensated with an entry to win one of four $500 CAD prepaid VISA cards.

##### Web panel sample

Given the low expected prevalence of online gambling in the general population, we sought an additional online sample recruited through a web panel. This online sample was intended to reach online gamblers more effectively and achieve adequate conceptual coverage [43]. Notably, however, these panels are typically made up of self-selecting participants, and as a result tend to overestimate people who gamble online and those who gamble more intensely [44].

Data were collected using a web panel (Léger Marketing, Montréal) over three periods (May to June 2021, July to August 2021, and September to October 2021). The sample was selected per quota to match the age, sex, region, and linguistic distribution based on census data. In total, 8,537 people out of 37,530 (22.7%) agreed to complete the online gambling eligibility screen. The final sample consisted of 3,295 participants excluding participants who were not eligible or did not complete the survey. As compensation, participants earned credits redeemable for gift cards on the survey firm’s website. The final survey sample consisted of 4,676 participants, including 4,376 current online gamblers and 300 online gambling quitters. Given that this study focused on current online gamblers, i.e., those who gambled during the first year of the pandemic, all post-pandemic online gambling quitters (*n*  = 300) were removed from the analyses exploring the impact of the pandemic. The sample comprised a majority of men (65.1%), people aged between 25 and 64 years (76.8%), with a secondary (36%) or college (38.4%) education, who were employed (66.9%), married or in a common law partnership (59.9%), and reporting an average annual household income of $30,000 to $59,999 (25.6%), with an overrepresentation of people reporting an income of $75,000 and more (49.4%).

#### Survey measures

##### Online gambling profiles

Respondents were asked to report their gambling participation during the past 12 months (i.e., during the first year of the COVID-19 pandemic and the implementation of public health measures) for nine online gambling activities (e.g., lotteries, bingo, slot machines, poker, casino games excluding poker, sports betting, esports betting, and day trading^1^), and during the 12 months preceding the pandemic.

Based on these questions, gamblers were assigned to one of the three profiles: (1) post-pandemic quitters are those who gambled online the year preceding the pandemic but did not report any online gambling activity during the first year of the pandemic, (2) current gamblers including both (a) new online gamblers, those who did not report any online gambling activity the year preceding the pandemic but gambled online during the first year of the pandemic, and (b) continuing online gamblers, those who reported online gambling the year preceding the pandemic and during the first year of the pandemic. To identify current online gamblers who migrated from offline to online gambling, participants were asked to report any offline gambling participation both during the first year of the pandemic and the year preceding it.

##### Problem gambling status

Participants completed the Problem Gambling Severity Index (PGSI), a validated questionnaire with reasonable temporal stability that is widely used to assess the frequency and severity of past-year gambling problems [45]. The PGSI consists of nine items that are scored from 0 to 3 (“Never”, “Sometimes”, “Most of the time”, “Almost always”) and summed. Respondents were categorized as: non-problem gamblers (score of 0), low-risk gamblers (1 or 2), moderate-risk gamblers (3 to 7), or problem gamblers (8+), based on the responses they provided for the past 12 months, which corresponds to the first year of the pandemic.

##### Impacts of the COVID-19 pandemic on gambling patterns

Respondents were asked to report whether (1) the frequency with which they spend money, (2) the amount of money they spend, and (3) the time they spend gambling has increased, decreased or remained unaffected by the COVID-19 pandemic for each of the gambling activities they reported betting on in the past 12 months.

##### Reasons for change in gambling behaviours during the pandemic

Respondents were asked if they consider that, since the start of the pandemic in March 2020, they gamble online less, as much as, or more than before the pandemic, or if they started or stopped gambling online during the pandemic. Based on their response, they were directed to a list of reasons where they can choose all the answers that apply. A similar list of nine reasons was provided to respondents who declared having started or increased their gambling online, with items referring to availability/safety, relational reasons (family, spouse), feelings of isolation, finances, social interaction, and relaxation/stress reduction. A similar list of nine reasons was provided to respondents who declared having stopped or reduced their gambling online, with items referring to not wanting to gamble around family members/children, feeling as though they gamble too much, financial strain, lack of interest, and mental health. Further questions queried respondents’ sex, self-identified gender, age, level of education, income, and work and marital status.

#### Weighting and analytical procedure

Survey data were weighted to adjust for non-response and the cluster sampling design, as well as to align the results with the information on the Québec adult population available from the census including age, sex, education, living conditions (living alone vs. living with others), and CMA. Estimates were produced separately for the web and telephone samples given significant differences in age distribution.

Since telephone- and web-based surveys potentially suffer from different methodological limitations in estimating online gambling prevalence in the population, we have employed two estimation methods based on weighted combinations of the telephone and web data: (1) an integration weight based on the proportion of individuals that were screened for eligibility in each of the two samples (65% telephone; 35% web), and (2) an integration weight that gave the telephone sample greater influence (80% telephone; 20% web). Jointly reporting the results of these intermediate scenarios, we seek to reduce sampling biases that may be present in each when reported individually.

For estimates among online gamblers, sampling weights were calculated jointly for the telephone and online samples to adjust for non-response and the clustered sampling design, as well as to align the results with the weighted distribution of gamblers according to the telephone sample, including age, sex, education, living conditions, CMA, and profile. Propensity scores were used in logistic regression to control for socio-demographic differences in the samples.

Analyses were conducted to estimate gambling prevalence and gambling problem severity among Québec adults and to verify whether these habits changed since the declaration of the COVID-19 pandemic. Estimates were produced with 5% confidence intervals. Descriptive analyses and Fisher’s chi square tests were performed to compare groups of online gamblers using Stata SE 16.1 (StataCorp; College Station, TX).

### Qualitative component

#### Sample selection and participants

After having completed the quantitative survey, participants were asked for their permission to be contacted to participate in the qualitative phase of the study; 2,277 agreed to be contacted (50.2%). A total of 415 participants who had gambled in the past 12 months were sent an invitation, and 98 interviews were scheduled. Of these, 96 semi-structured interviews were conducted. The sample was formed using the non-probabilistic maximum variation sampling method [46, 47], based on the following diversification criteria that are recognized as having a potential impact on the phenomenon under study: trajectories with online gambling (continuing, migratory and new online gamblers), direction of the changes in online gambling habits due to the pandemic (increase, decrease and no change), gambling activities (controlling exclusive lottery), gender and age group. The criteria were established based on self-reported responses to the survey. Among these profiles, over half (*n* = 58) reported having increased their gambling habits during the pandemic, 17 reported having decreased them, 16 mentioned that their gambling habits remained stable, and a small proportion were new gamblers (*n* = 5).

#### Interview grid and procedure

The interview grid explored (1) the online gambling habits (e.g., practices, benefits, harms, motivations) and their variations over the past 12 months; (2) the impact of the pandemic and health measures on online gambling practices (turning points, benefits, harms, reasons for changes, etc.); (3) the use of responsible gambling measures; and (4) and the use of help services. However, this paper reports on the impact of the pandemic on online gambling practices only. 

Gambler’s lived experiences during the pandemic were explored via semi-structured interviews which took place between July and November 2021 via Zoom and lasted approximately 1.5 h. After having presented each participant with the consent form and received their verbal consent to participate in the study, the interview was conducted by one of six interviewers (i.e., one of the principal researchers and five research assistants who were in graduate programs in the fields of psychology, sociology and health science). The final qualitative sample was composed of 37 participants who identified as women and 59 as men. The mean age of the group was 48 years, within a range from 18 to 82 (*SD*  = 1.4); 20% of participants were aged between 18 and 34 and 40% between 35 and 54. Approximately 68% of participants were in a relationship (married, common law partnership) and 26% were single. A small proportion of participants (17.7%) lived alone. The vast majority (84%) had a post-secondary diploma. Just over half of the sample (56.3%) was employed. The median annual household income was between $75,000 and $99,000, and 13% had an income of less than $30,000.

Participants in the qualitative study reported an average of 2.5 online gambling activities (min = 1, max = 7, *SD* = 1.4). Three quarters bought lottery tickets online, and 43.5% purchased scratch tickets. Just over half played online casino slots and almost 22% played other online casino games. Nearly 18% played online poker and around 15% practiced day trading. Finally. around 15% of participants reported taking part in online sports betting, 12% in online bingo, and 2% in esports betting.

#### Data analysis

The interview content was recorded in audio format, then transcribed with the aid of automatic transcription software and validated by a research assistant. NVivo 12 software was used to carry out thematic content analysis on the data by following the method proposed by Paillé and Muchielli [48]. The themes were identified using a mixed approach, i.e., according to pre-established themes drawn from the interview guide (deductive), and according to themes that emerged directly from the participants’ words (inductive). The codebook was developed in close collaboration between the two principal researchers and the research professional responsible for the analyses. Bi-monthly meetings were held throughout the process to discuss content coding and theme development. Review and updating of the codebook continued on a regular basis throughout the analysis process to ensure the credibility of the analyses (internal validity) [46, 49].

## Results

### Online gambling prevalence

The prevalence of online gambling during the first year of the pandemic was estimated at 10.9% in the telephone-based sample and 37.3% among the sample derived from a web panel. When the two databases were integrated, the prevalence of online gambling was estimated at 15.6% when allocated weights for telephone and web data were 65% and 35% respectively, and 20.3% when the weights were 80% and 20% (Table 1). Using the latter integration weights, the data revealed that 1.2% of people reported having quit online gambling during the first year of the pandemic whereas 5.1% were new online gamblers who either started gambling during the first year of the pandemic (2.1%) or migrated from offline gambling activities to online formats (2.8%).

**Table 1:** Prevalence of online gambling during the first year of the COVID-19 pandemic

	Weight doubled for telephone sample			Proportional weight for both samples		
	(*n* = 24,354)			(*n* = 24,354)		
	%	CI (95%)	Pop. est.	%	CI (95%)	Pop. est.
Lifetime non online gamblers	77.1	76.4–77.8	4 918 883	71.3	70.6–72.0	4 547 249
Online gambling quitters	7.3	6.8–7.7	462 217	8.4	8.0–8.8	534 615
Quit before the pandemic	6.0	5.6–6.5	385 429	7.0	6.6–7.4	444 957
Quit during the pandemic	1.2	1.0–1.4	76 787	1.4	1.2–1.6	89 659
Current online gamblers (past 12 months)	15.6	15.0–16.3	996 446	20.3	19.7–21.0	1 294 281
New online gamblers	5.1	4.7–5.5	324 426	5.6	5.2–6.0	355 153
Reported gambling for the first time	2.1	1.9–2.4	135 660	2.3	2.0–2.6	144 205
Migratory gamblers (offline to online)	2.8	2.5–3.1	176 638	3.1	2.9–3.4	197 652
Continuing gamblers	10.5	10.0–11.0	672 020	14.7	14.2–15.3	939 127

The findings revealed that new and migratory online gamblers were more likely to be exclusive lottery players and less likely to report betting on scratch tickets and instant lotteries, sports and poker (Table 2). New gamblers were most likely to report engaging in day trading whereas migratory gamblers were the most likely to report betting on bingo. There was a significant difference between the three groups in the proportion of gamblers who gambled on lottery tickets and slot machines, with the lowest percentages having been reported by new gamblers and the highest among continuing gamblers.

Continuing gamblers reported betting on more gambling activities than new and migratory gamblers; they also exhibited a higher proportion of moderate- and high-risk gamblers.

**Table 2:** Gambling activities and gambling problem severity among online gamblers during the first year of the COVID-19 pandemic

	Current gamblers		New gamblers	Migratory gamblers	Continuing gamblers					
	(*n* = 4,376)		(*n* = 465)	(*n* = 679)	*(n* = 3,232)					
	%	CI (95%)	%	%	%	Χ^2^ (df)	*p*			
**Gambling activities**										
Lottery	77.2	75.9–78.4	63.2^a^	76.3^b^	79.5^b^	63.43(2)	< 0.001			
Lottery tickets	71.1	69.8–72.5	57.0^a^	65.5^b^	74.3^c^	72.82(2)		< 0.001		
Scratch tickets and instant lottery	37.6	36.1–39.0	25.8^a^	32.0^a^	40.4^b^	48.66(2)		< 0.001		
Slot machines	27.1	25.7–28.4	15.6^a^	21.7^b^	29.8^c^	53.59(2)		< 0.001		
Sports betting	16.2	15.1–17.3	10.3^a^	7.5^a^	18.7^b^	63.05(2)		< 0.001		
Table games	12.9	11.9–13.9	7.6^a^	11.9^ab^	13.8^b^	15.04(2)		< 0.001		
Day trading	12.5	11.5–13.5	19.7^a^	7.7^b^	12.3^c^	36.19(2)		< 0.001		
Poker	12.0	11.1–13.0	6.3^a^	6.8^a^	13.9^b^	43.57(2)		< 0.001		
Bingo	7.6	6.8–8.4	6.8^ab^	3.9^a^	8.4^b^	15.85(2)		< 0.001		
Esports betting	3.3	2.8–3.9	3.6^a^	1.3^b^	3.7^a^	8.49(2)		0.014		
Lottery tickets only	26.7	25.4–28.0	30.6^a^	36.1^a^	24.2^b^	42.83(2)		< 0.001		
	M	CI (95%)	M	M	M					
Number of online games	2.01	1.97–2.05	1.54^a^	1.60^a^	2.16^b^	*F* = 87.79(2)			< 0.001	
**Gambling problem severity**						**36.53(4)**			**< 0.001**	
Non problem/Low risk	81.5	80.3–82.7	86.9^a^	87.9^a^	79.5^b^					
Moderate risk	10.8	9.9–11.8	8.3^a^	6.2^a^	12.1^b^					
High risk	7.7	6.9–8.5	4.8^a^	5.9^a^	8.4^b^					
**Gambling problem severity** **(excluding lottery players only)** (***n*** **= 2**,**899)**						**23.66(4)**				**< 0.001**
Non problem/Low risk	76.2	74.6–77.8	81.4^a^	83.7^a^	74.3^b^					
Moderate risk	13.8	12.6–15.1	11.8^a^	7.3^b^	15.1^a^					
High risk	10.0	8.9–11.1	6.8^a^	9.0^ab^	10.6^b^					

Note. Superscript letters differing between two columns indicate statistically significant differences between groups (*p <* 0.05)

### Impacts of the pandemic on gambling habits

The findings revealed that continuing gamblers reported similar impacts on the frequency, spending and time for the various gambling activities (Fig. 1a, 1b and 1c). The impact of the pandemic seems to have been the least remarkable for lottery games and the most for day trading, tables games and slot machines. Esports and sports betting are the activities for which participants reported the largest decreases in their gambling frequency, expenditure, and time.

**Fig. 1a:**
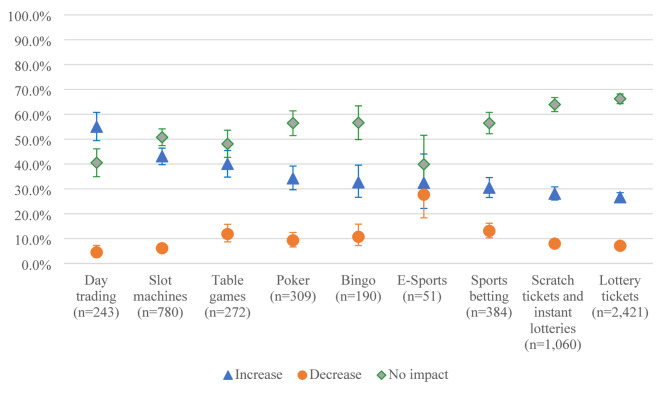
Impacts of the COVID-19 pandemic on gambling frequency according to type of gambling activity (*n* = 3,232)

**Fig. 1b:**
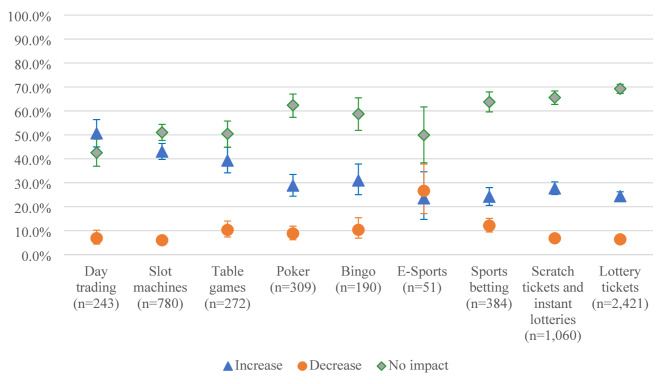
Impacts of the COVID-19 pandemic on gambling expenditure according to type of gambling activity (*n* = 3,232)

**Fig. 1c:**
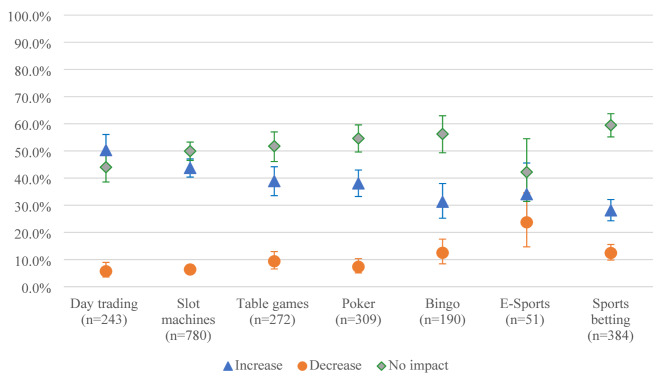
Impacts of the COVID-19 pandemic on time spent gambling according to type of gambling activity (*n* = 3,232)

### Reasons for stopping or decreasing online gambling during the pandemic

**Fig. 2:**
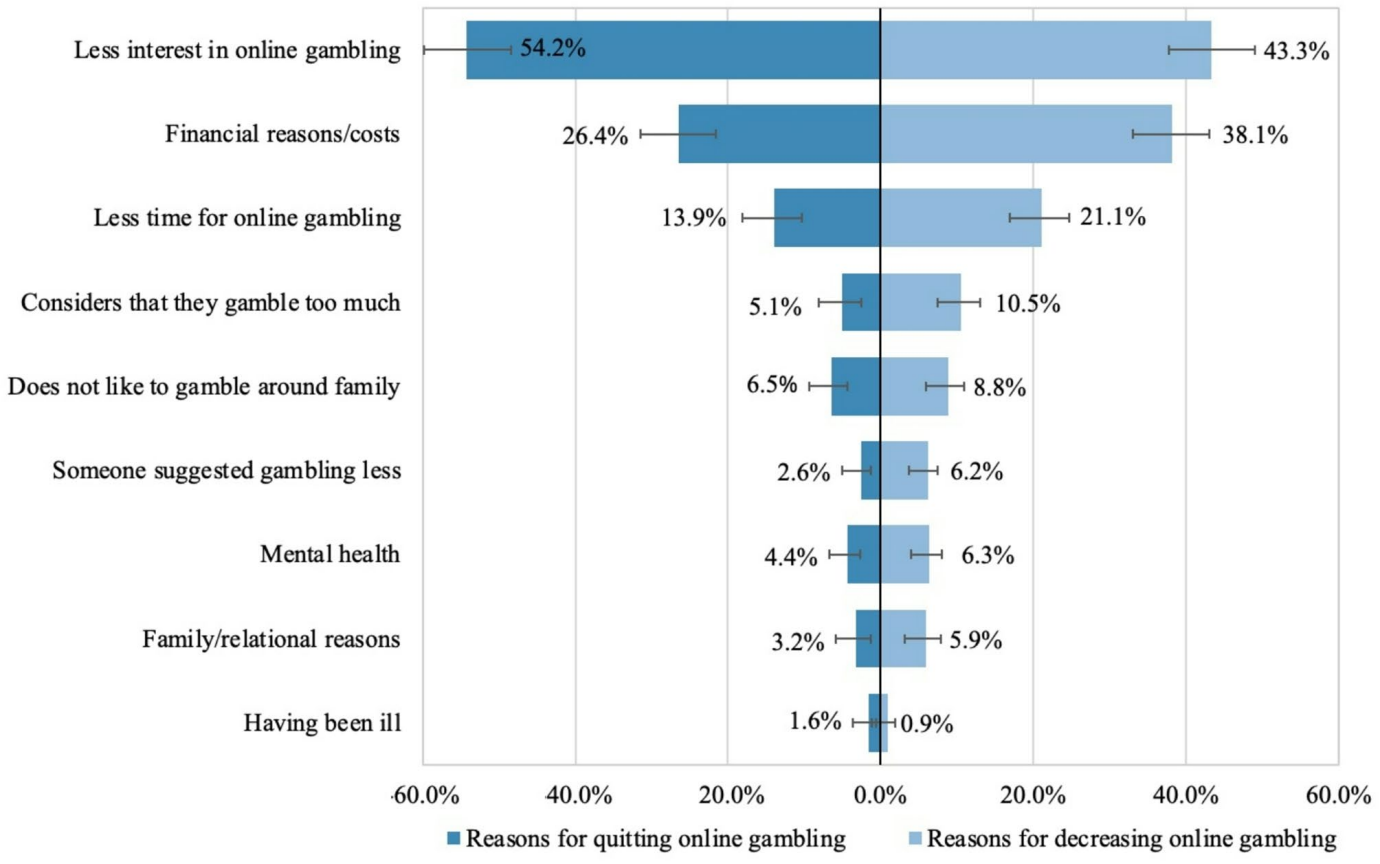
Reasons for reducing or quitting online gambling during the COVID-19 pandemic^1^ ^1^The sample included 369 online gamblers who declared having reduced their gambling due to the pandemic, and 300 who quit gambling

Many reasons were reported by online gamblers to explain the changes in their online gambling habits during the pandemic. Among participants who stopped or decreased their online gambling during the pandemic, the most reported reason was being less interested in online gambling (54.2%) to a higher extent among those who stopped gambling online, followed by reasons related to the cost of online gambling, particularly among those who decreased their gambling, and having less time (Fig. 2). To a lesser extent, online gamblers reported reasons related to not wanting to gamble around family members, feeling that they gambled too much or being told so, and mental health.

The qualitative interviews conducted with online gamblers having reduced their participation during the pandemic highlighted that in some cases, spending too much time gambling online was a reason for decreasing online gambling engagement. Alice described how she felt that she spent too much time gambling, which she described as a practice that became “a bit unhealthy”, even risky:

*I also had the impression that it was becoming almost a bit unhealthy*,* because we were doing it more to pass the time and I was afraid of getting in too deep*,* not into an addiction*,* but I don’t know if you understand a bit about that. Instead of being just a bit of fun from time to time*,* it was becoming too much of something to pass the time*,* to relieve boredom*,* and then really spending too much time doing it?* (Alice – Sports betting, poker, slot machines)

Some others, such as Brad, reported that the pandemic constituted an opportunity to reflect on one’s financial priorities and refocus them on other types of activities:

*There’s not*,* there’s been a little difference. Before that*,* I was like a big*,* not a big gambler*,* but an average online gamer*,* all that. But with the pandemic*,* I kind of thought that the money I had*,* I had less to spend on gambling and stuff. I’ve kind of cut back a lot on that. Before that*,* especially with Loto-Québec gambling sites*,* it was easy*,* and you could go there every day. But with the pandemic*,* I concentrated on my volunteer work*,* my kids and all that. It took my mind off gambling.* (Brad – Sports betting)

### Reasons for starting or increasing online gambling during the pandemic

**Fig. 3:**
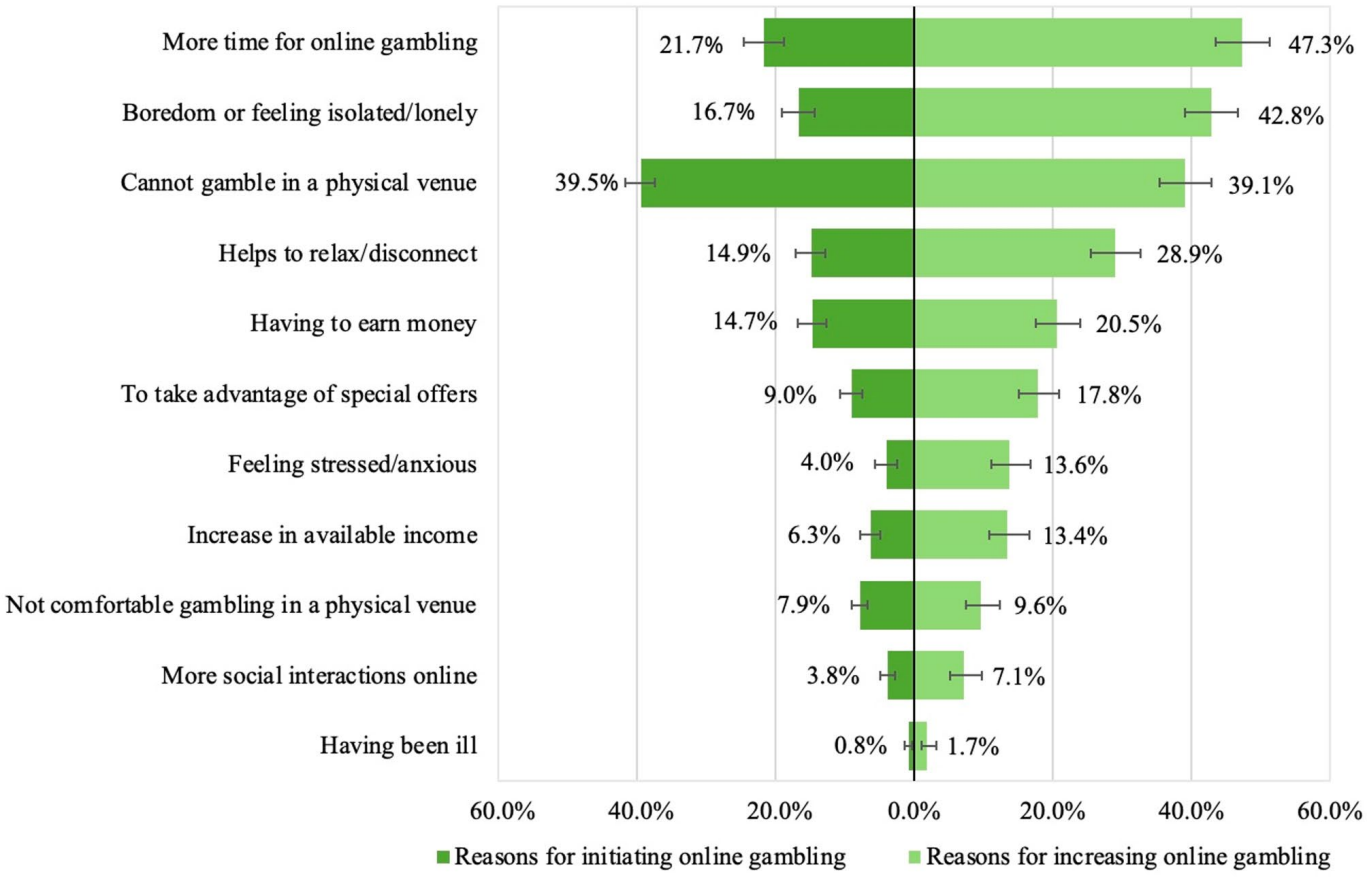
Reasons for starting or increasing online gambling during the COVID-19 pandemic.^2^ ^2^The sample included a total of 1,144 initiators (465 new gamblers and 679 migratory gamblers) and 962 online gamblers who declared having increased their gambling due to the pandemic

The most reported reasons by new gamblers for starting to gamble online was not being able to gamble in a physical venue, a reason that also seems to have been important for gamblers who increased their betting online (Fig. 3). For continuing gamblers, having more time was the most reported reason for gambling more online. For new gamblers and gamblers who increased their betting online, feelings of boredom and isolation, and the need to relax and disconnect were also among the top reasons underlying change in their online gambling habits, followed by the need to earn money. Most reasons were reported proportionally more by continuing gamblers having increased their gambling participation than new gamblers.

The qualitative interviews conducted with online gamblers offered personal points of view as to the reasons why some of them came to start gambling online during the pandemic. Many participants mentioned that they began gambling online due to an inability to do so offline due to public health restrictions, which was the case of Nicolas who sought a way to be able to continue gambling when it became impossible for him to buy scratch tickets:

*Yes. exactly. Then probably. I’m pretty sure too. it’s because I said to myself: “well*,* it’s been a long time since I’ve been out*,* since I’ve bought a scratch ticket*,* so I’ve been looking for a way to play*,* and that’s that.* (Nicolas – Lottery, scratch tickets and instant lottery, slot machines)

Other participants evoked the fact that they had more time on their hands during the pandemic to spend gambling online. For instance, Greg described his experience with day trading as a unique event which would not have happened without the pandemic because he had much more free time to dedicate to this new activity:

*In fact*,* I discovered the stock market*,* let’s say the stock market*,* day by day*,* you know*,* I discovered*,* let’s say every day. I was keeping track of it. So*,* without the pandemic*,* I would probably never have taken up the stock market day by day. I think the pandemic led me to take an interest. Otherwise*,* I don’t think I would have seen a business opportunity in this field one day. (…) I clearly would have had less time*,* and without that I’d be working for (company) today*,* and I don’t even know if I’d be aware of what’s happening on the stock market. So it’s had quite an impact all the same.* (Greg – Day trading)

Participants who spoke of an increase in their online gambling habits during the pandemic reported that online gambling was a way for them to alleviate the emotional burden that the pandemic had prompted. Maxime described such an experience while referring to the “void” caused by the peak phase of the pandemic:

*Well*,* in the beginning it was a soothing balm for everything that was going on*,* as opposed to before*,* otherwise no. I didn’t… It filled a void during the peak of the pandemic.* (Maxime – Lottery, scratch tickets and instant lottery, slot machines, poker, day trading)

Among those who had increased their online gambling habits during the pandemic, some reported having increased the amount of money they spent on online gambling since they hoped that doing so would allow them to earn money which they lacked due to changes in their work situation. This was the scenario that Brigitte experienced:

*Yes*,* it’s foolish*,* but yes*,* because I had a smaller income since I was working less. The lure of money made me want to catch up*,* but it had the opposite effect.* (Brigitte – Lottery, slot machines)

## Discussion

This study aimed to capture the impact of the COVID-19 pandemic and the related public health measures on online gambling practices in the adult population of the province of Québec, Canada. The results revealed several important trends. Foremost among them is the fact that many Québec residents who had not previously participated in online gambling chose to do so for the first time or had increased their engagement in this form of activity during the first year of the pandemic.

For a considerable proportion of current gamblers, these trends appear to have stemmed from the unavailability of land-based gambling venues (e.g., casinos and bars) that were closed on provincial health orders, from having more free time, or for emotional reasons (i.e., to relieve stress or boredom). Some others, however, reported that a need to earn money was one of the motivations that fueled such changes in their gambling behaviours. This reason was also documented in other studies having explored the motivations underlying increases in gambling habits during the pandemic [6, 7, 37, 38]. Financial need is a plausible reason that may have motivated gambling behaviour given the economic instability that prevailed in Québec during the pandemic. Indeed, health and social measures were among the most severe in the world (i.e., lockdown, curfew), undermining jobs and the financial situation of citizens. Thus, this may have brought some gamblers to consider online gambling as one potential solution to financial constraints.

One of the crucial questions that this finding raises pertains to why gambling is still perceived as a way to making money among gamblers given that most interventions among gamblers have been focused on deconstructing this popular belief, often referred to as cognitive distortions. It is our contention that, beyond an individually focused understanding of this finding, one should also reflect on how the parameters of the gambling ecosystem, such as the ways in which gambling products are commercialized, may influence the persistence of such popular beliefs. Our own study has shown that gamblers referred to gambling industry practices and their promotions and advertisements as being some of the reasons why they started gambling online or persisted or increased their involvement in it [38].

From a public health perspective, it is difficult to fathom why most jurisdictions have been silent about regulating the industry practices for a popular form of consumption such as gambling that is ‘no ordinary commodity’ [50]. In many countries, the online gambling market remains unregulated or, at best, regulated as a financial market. From a public health perspective, online gambling will remain an important issue as is shown through multiple ongoing debates on regulation happening in most western countries, including Canada. It is our contention that, moving forward, such a debate (1) should be conducted nationally and as part of an international concerted strategy, (2) should occur independently from the governmental institutions and gambling operators, which can only happen when an independent body is leading such an initiative, and (3) should put public health priorities at the forefront of the debate [51].

The COVID-19 pandemic is a rare phenomenon that revealed unique data on gambling practices during critical periods of adversity for people and the society they live in. While some people may not have been interested in starting or migrating to online gambling during the pandemic, thus potentially solving gambling-related issues at least temporarily, our study revealed that in such periods, some people turned to online gambling to compensate for failing social interactions and to mitigate stress, anxiety, and boredom. It also revealed that these forms of gambling are particularly harmful and are associated with more loss of control and problems. Hence, such results point to the importance of prioritizing and dedicating resources to researching novel digital ways for reaching out to isolated online gamblers. New technologies and the availability of big data on online platforms provide an optimal space to push forward tools for detection and interventions among online gamblers. Although promising artificial intelligence-based detection models have been more visible in the past years [52–54], the transfer of these models into useful and efficient tools will require a concerted effort to ensure their ethical and fairness foundations. To achieve this end, resources and interdisciplinary collaborations are paramount.

The major contribution of this study lies in its representative nature of its sample, hence providing an extensive portrait of the impact of the pandemic on adult online gamblers. Another strength of this study resides in its mixed methods approach that enabled a more comprehensive analysis of the impact of the health crisis on online gambling practices, while merging the quantitative populational snapshot with information on the lived experiences of gamblers. Still, the study has some limitations to consider, including the potential for participant under-reporting of gambling involvement, as noted elsewhere [38], especially among individuals heavily involved in gambling. Also, despite the strength of having a randomly selected sample for the telephone survey, the response rate was expectedly low, which could limit the representativity of the sample. By adding a web panel sample, this study aimed to provide statistical strength as well as to gain access to people who are likely to be more active on the Internet, including to gamble online.

## Conclusion

Addiction researchers anticipated that pandemic-related public health measures would exacerbate an increasing trend in online gambling and initiate a migration of gamblers towards online platforms that typically see a high rate of people experiencing harms [26]. This is what has occurred in Québec. The full impacts of this event will be revealed in coming years, including a 72% increase in online gamblers seeking to exclude themselves from accessing Québec's provincial gambling website in 2020 [55]. The steady increase in online gambling markets makes it even more urgent to put the issue back onto the public health agenda. Like a magnifying glass, the pandemic has enabled us to see more clearly the complexity and interrelatedness of the multiple determinants of online gambling behaviour. Of diverse and multi-levelled nature, these factors touch on individual dimensions of isolation, distress and boredom, as well as ecological factors relating to the supply and promotion of games. Thus, the online gambling ecosystem calls for preventive actions that concern the individual, the gambling offer and the environment in which this offer is deployed. Public policies, which are powerful tools for online gambling market regulation, are needed. They must be accompanied by promising tools for the detecting and prevention of online gambling-related harms, and public resources for gambling treatment services should be allocated to meet the potential influx of people experiencing harms associated with online gambling. The scale of these needs will become clearer as researchers continue tracking these trends for years to come.

## Data Availability

No datasets were generated or analysed during the current study.

## Footnotes

1 While day trading is not inherently a form of gambling activity, its structural characteristics share some similarities with gambling. The speculative nature and short timeframe of day trading make it a high-risk form of trading activity. It is also a form of trading which can attract individuals who tend to be sensation-seekers and risk-takers. This comparison has been made in both the fields of behavioral finance and gambling.
